# Examining the Effectiveness of Polyamidoamine (PAMAM) Dendrimers for Enamel Lesion Remineralization: A Systematic Review

**DOI:** 10.7759/cureus.64490

**Published:** 2024-07-13

**Authors:** Sri Meghana Sanka, Kavitha Ramar

**Affiliations:** 1 Pediatric and Preventive Dentistry, Sri Ramaswamy Memorial (SRM) Kattankulathur Dental College and Hospital, Chennai, IND; 2 Pedodontics and Preventive Dentistry, Sri Ramaswamy Memorial (SRM) Kattankulathur Dental College and Hospital, Chennai, IND

**Keywords:** a systematic review, dendrimers, enamel remineralization, remineralizing agents, surface microhardness

## Abstract

White spot lesions (WSLs) and demineralized enamel surfaces are common dental issues that can lead to further complications if untreated. The potential of various remineralizing agents has been extensively studied, but the efficacy of polyamidoamine (PAMAM) dendrimers in promoting enamel remineralization remains to be fully elucidated. This systematic review aims to evaluate the remineralizing potential of PAMAM on WSLs and demineralized enamel surfaces. To identify relevant studies, a comprehensive literature search was conducted across multiple databases, including PubMed, Scopus, and Cochrane Library. Inclusion criteria comprised in vitro and in vivo studies that assessed the effects of PAMAM on WSLs or demineralized enamel. Data extraction and quality assessment were performed independently by two reviewers. The primary outcomes measured were changes in enamel microhardness, surface morphology, and mineral content. Five studies met the inclusion criteria, comprising in vitro studies. The results indicated that PAMAM demonstrated a significant remineralizing effect on demineralized enamel surfaces, as evidenced by increased microhardness and improved surface morphology. The studies varied in their methodological approaches but collectively supported the potential of PAMAM in enamel remineralization. PAMAM dendrimers exhibit promising remineralizing properties for treating WSLs and demineralized enamel surfaces.

## Introduction and background

Dental caries is a widespread oral health issue that results in the demineralization of enamel, leading to cavities and tooth decay [1]. White spot lesions (WSLs) are the initial clinical indication of enamel demineralization and are among the most noticeable clinical indicators that precede the start of caries [2]. Because of the increased porosity of the enamel, they seem opaque and chalky [2]. WSLs cause reversible lesions if detected early. On the other hand, WSLs may develop into cavitated lesions if the acidic environment is not removed or diminished [3]. Enamel remineralization is a crucial process in reversing early carious lesions and preventing further decay [3]. Over the years, various remineralizing agents have been developed and studied, including fluoride [4], casein phosphopeptide-amorphous calcium phosphate (CPP-ACP) [5], and hydroxyapatite, among others [6].

Dendrimers/polyamidoamine (PAMAM) are a relatively new class of nanomaterials characterized by their highly branched, treelike structure [7]. These synthetic macromolecules have unique properties, such as a high degree of surface functionality and precise molecular weight, which make them suitable for various biomedical applications, including drug delivery, imaging, and therapeutic agents [8]. Recent studies have suggested that dendrimers may possess significant potential in dental applications, particularly in enamel remineralization [8]. This systematic review and meta-analysis aimed to evaluate the remineralization potential of dendrimers on enamel in permanent teeth. By comparing the efficacy of dendrimers to other established remineralizing agents, this study seeks to provide a comprehensive assessment of their potential use in clinical practice.

## Review

Methods

Search Strategy

**Figure 1:**
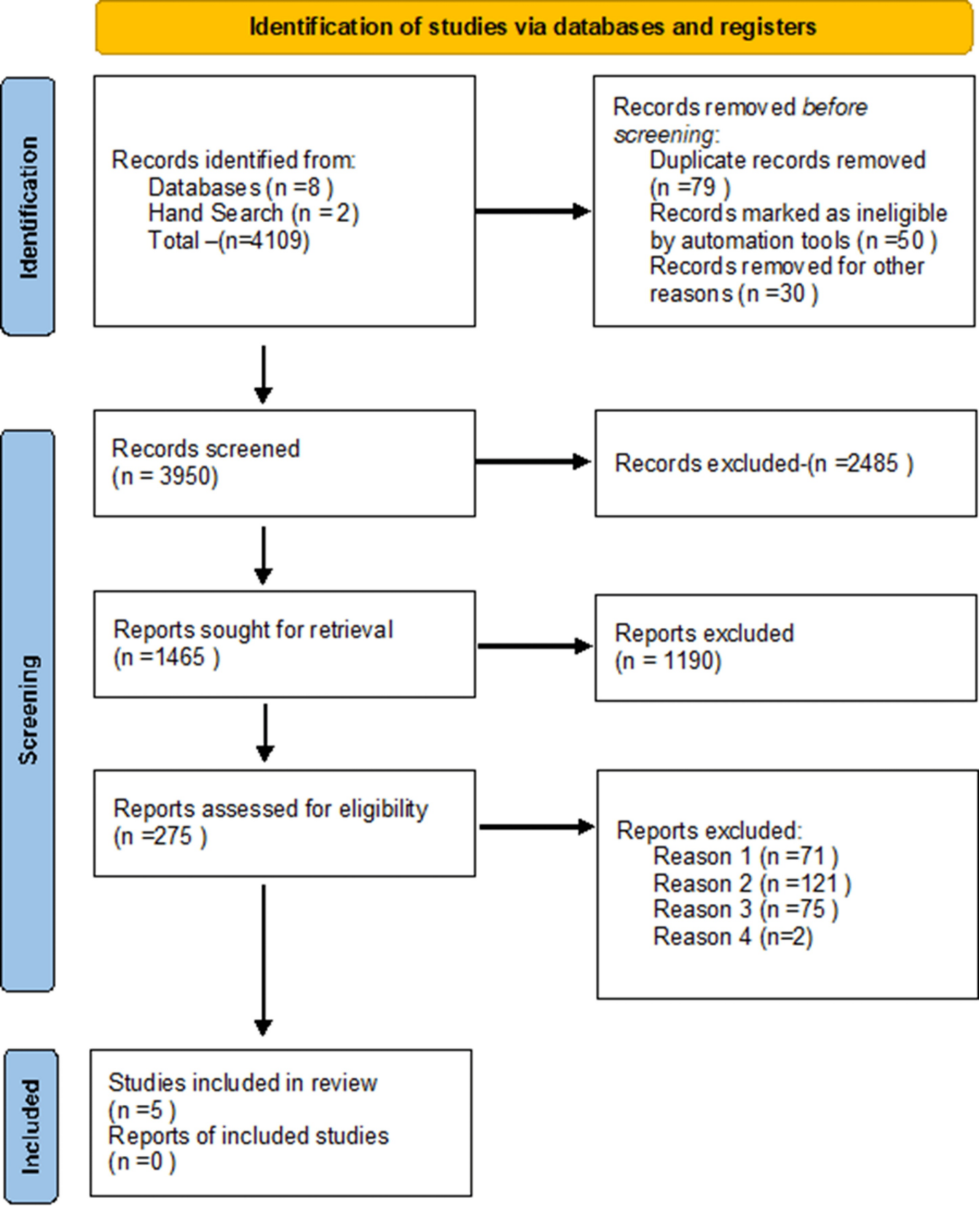
PRISMA diagram detailing the study identification and selection process PRISMA: Preferred Reporting Items for Systematic Reviews and Meta-analyses

The Preferred Reporting Items for Systematic Reviews and Meta-analyses (PRISMA) statement for reporting systematic reviews recommended by the Cochrane Collaboration was followed for conducting this systematic review (Figure 1). PubMed, Google Scholar, CENTRAL, and Embase were searched for peer-reviewed research published till June 2024. Databases were searched using the search terms under two search themes and combined using the Boolean operator "AND," "OR," and "NOR." The search strategy incorporated various combinations of keywords and medical subject headings (MeSH) terms related to dendrimers, enamel, and remineralization. The following keywords and their combinations were used: "dendrimers," "enamel remineralization," "dental caries," "permanent teeth," and "remineralizing agents" [7,8].

Selection Criteria

Studies involving the permanent or primary teeth, studies examining the use of dendrimers for enamel remineralization, and studies comparing dendrimers with other remineralizing agents were included. Randomized controlled trials (RCTs), clinical trials, in vitro studies, and in vivo studies were also included. Studies without PAMAM as a remineralization agent were excluded. Studies not reporting relevant outcomes and reviews, case reports, editorials, and ex vivo studies were also excluded.

Data Abstraction

The systematic review process began with a comprehensive literature search conducted across multiple electronic databases, including PubMed, Scopus, and the Cochrane Library, to identify studies evaluating the remineralization potential of dendrimers on enamel [9]. From this initial search, a total of 4109 articles were identified. After removing duplicates and excluding studies in other languages, 3950 unique records remained for screening. In the first phase of screening, the titles and abstracts of these 3950 records were reviewed independently by two reviewers to assess their relevance based on the predefined inclusion and exclusion criteria. Studies were included based on inclusion criteria. This screening process resulted in the exclusion of 3675 studies, leaving 275 full-text articles for further assessment.

The full-text articles were then independently reviewed by the same two reviewers. During this stage, studies were meticulously examined to confirm that they met all inclusion criteria and to extract detailed information about their methodologies, interventions, comparisons, and outcomes. Any discrepancies between the reviewers were resolved through discussion and consensus, with a third reviewer consulted if necessary. This thorough evaluation led to the exclusion of 269 studies, primarily due to reasons such as inadequate data reporting, inappropriate study design, or failure to meet the inclusion criteria (Figure 1). Ultimately, five studies were deemed eligible and included in the qualitative synthesis. This systematic review has been registered in PROSPERO CRD42024539616, and we have followed the PRISMA statement [10]. Data has been extracted using a standardized data extraction form, which will include the following information: study characteristics (author, year, country, study design), comparison details (type of remineralizing agent), and outcome measures (remineralization potential, measured through various techniques such as surface microhardness, scanning electron microscopy (SEM), and others).

Quality Assessment and Data Analysis

Two reviewers (SM and KR) independently assessed each article using the Cochrane Handbook for Systematic Reviews [11]. The quality of the studies included will be assessed using the Quality Assessment Tool for In Vitro Studies (QUIN) tool. Each domain was given a risk-of-bias (ROB) rating of low, high, or unclear. Following that, each study's degree of risk was graded as low (all domains met), moderate (one or two domains not met), or high (all domains not met) (three or more domains not met) [12]. Meta-analysis was not possible due to the heterogeneity of the studies.

Outcomes

Study Characteristics

In the present review, all five studies were in vitro studies and were compared based on treatment methods, study design, and outcome of the studies. Three studies have compared their results using scanning electron microscope and X-ray diffraction method. The rest of the studies were compared using Vickers hardness test results (Table 1).

**Table 1:** This table represents the key characteristics of the included studies SEM-EDX: Scanning electron microscopy with energy dispersive X-ray (EDX); COOH-PAMAM: carboxyl-terminated polyamidoamine dendrimer; NH2-PAMAM: amine-terminated polyamidoamine dendrimers; SN15 PAMAM: salivary statherin protein-inspired polyamidoamine; PAMAM: polyamidoamine; NACP: amorphous calcium phosphate; CPP-ACPF: casein phosphopeptide-amorphous calcium phosphate with fluoride

Title	Author	Journal	Year of the study	Type of study	Type of analysis done	Generation of PAMAM	Comparative group
Remineralization effectiveness of the PAMAM dendrimer with different terminal groups on artificial initial enamel caries in vitro	Fan et al. [13]	Dental Materials	2019	In vitro	Vickers hardness test, SEM-EDX	COOH PAMAM	NH2 PAMAM, OH-PAMAM
Enamel remineralization via poly(amido amine) and adhesive resin containing calcium phosphate nanoparticles	Gao et al. [14]	Journal of Dentistry	2019	In vitro	Vickers hardness test, SEM-EDX	SN15 PAMAM	NACP
The effect of poly amido amine dendrimer, nano-hydroxyapatite and their combination on microhardness of demineralized enamel	Khater et al. [15]	Al-Azhar Journal of Dentistry	2024	In vitro	Vickers hardness test, SEM-EDX	COOH-PAMAM	Nanohydroxyapatite
A comparative assessment of remineralization potential of sodium fluoride (Naf) and poly amido amine (Pamam) on artificial caries like lesion of enamel-an in vitro study	Deokar et al. [16]	Medicon Dental Science	2023	In vitro	Vickers hardness test, SEM-EDX	NH2-PAMAM	Sodium fluoride
In vitro comparison of enamel remineralization in primary teeth using polyamino amine vs. casein phosphopeptide-amorphous calcium phosphate fluoride	Meghana et al. [17]	IJDSIR	2024	In vitro	Vickers hardness test, SEM-EDX	PAMAM	CPP-ACPF

The included in vitro studies have "dendrimers" as the test group and "other remineralizing agents" as the comparator. This ROB assessment aims to evaluate the ROB in invitro studies. The outcome assessed was the remineralization potential of Dendrimers on enamel.

Each in vitro study was evaluated for ROB by two reviewers (SM and KR) using the QUIN tool [18] designed specifically for dental-based in vitro studies. This instrument consists of twelve main domains and an overall ROB domain (based on the final score). The 12 main criteria are clearly stated aims and objectives, detailed explanation of sample size calculation, detailed explanation of sampling technique, details of the comparison group, detailed explanation of methodology, operator details, randomization, method of measurement of outcome, outcome assessor details, blinding, statistical analysis, and presentation of results. These criteria are scored as either as anyone: adequately specified (score 2), inadequately specified (score 1), and not specified (score 1). Criteria seven and nine were excluded from this ROB assessment, as it was found to be "not applicable" for the in vitro studies included in this systematic review. The final score was calculated using a formula.

**Figure 2:**
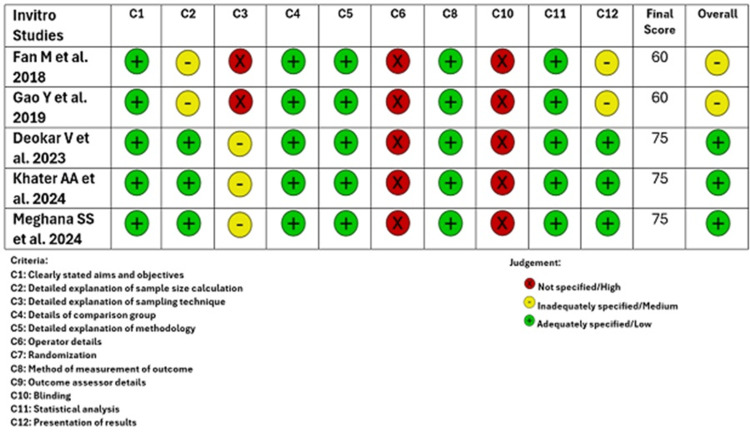
Traffic light plot-QUIN tool assessment of the included studies (criteria seven and nine are excluded for this ROB analysis of in vitro studies) QUIN: Quality Assessment Tool For In Vitro Studies; ROB: risk of bias Fan et al. [13], Gao et al. [14], Khater et al. [15], Deokar et al. [16], Meghana et al. [17]

**Figure 3:**
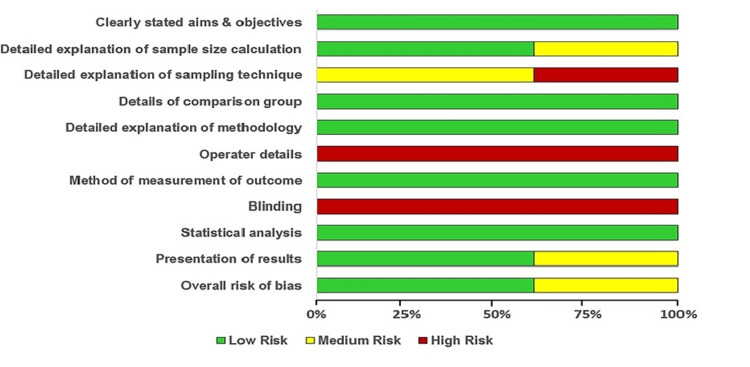
Summary bar plot-QUIN tool assessment of the included studies (criteria seven and nine are excluded for this ROB analysis of in vitro studies) QUIN: Quality Assessment Tool For In Vitro Studies; ROB: risk of bias

If the final score (overall ROB) for each of the included in vitro studies (n = 5) was >70%, then the particular in vitro study was graded as low ROB. A score between 50% and 70% was graded as a medium ROB, and <50% was a high ROB. Disagreements regarding the same were settled by the second reviewer (KR). The 10 criteria-level judgments for each in vitro study (traffic light plot) and the distribution of ROB judgments within each bias criterion (summary bar plot) were generated to represent the ROB assessment graphically using MS Excel (Microsoft Corporation, Redmond, Washington, United States) (Figures 2 and 3). Figure 2 shows, among the included studies (n = 5), that three studies have a low overall ROB and two studies have a medium overall ROB. Figure 3 depicts a 60% low bias and a 40% medium overall ROB.

Discussion

The present systematic review aimed to evaluate the remineralization potential of dendrimers on enamel compared to other remineralizing agents. The findings of this review provide important insights into the effectiveness of dendrimers as a novel therapeutic approach for enamel remineralization. The analysis of the included studies demonstrated that dendrimers exhibit significant potential in enhancing enamel remineralization [18]. The unique branched structure of dendrimers, which allows for high functionalization and multivalency, likely contributes to their superior performance. These properties facilitate the delivery of active remineralizing agents to the enamel surface, promoting the formation of hydroxyapatite crystals and improving mineral density [19].

Mechanism of Action

The mechanism by which dendrimers enhance remineralization appears to involve multiple pathways [19]. The high density of functional groups on the dendrimer surface can chelate calcium ions, promoting their deposition on demineralized enamel. Additionally, the presence of these functional groups may facilitate the binding of phosphate ions, further contributing to hydroxyapatite formation. Some studies also suggest that dendrimers can modulate the local pH environment, creating conditions that are favorable for remineralization [20].

Clinical Implications

The clinical implications of these findings are significant. The ability of dendrimers to effectively remineralize enamel positions them as a promising adjunctive treatment in preventive dentistry. Their use could potentially reduce the incidence of caries and enhance the longevity of dental restorations [20]. Moreover, dendrimers' ability to penetrate deeper into the enamel could make them particularly useful in treating early carious lesions and WSLs, which are challenging to manage with conventional remineralizing agents [21].

The study by Fan et al. [13] aimed to evaluate the remineralization effectiveness of PAMAM with different terminal groups on artificial initial enamel caries in vitro. Enamel specimens were prepared and demineralized to create artificial initial caries lesions, then divided into groups based on the terminal group of the PAMAM dendrimers: amine-terminated, carboxyl-terminated, and hydroxyl-terminated. Each group was treated with its respective PAMAM dendrimer solution. The remineralization effectiveness was assessed using microhardness testing and SEM with energy dispersive X-ray (EDX) analysis . Results showed that all PAMAM dendrimer groups exhibited a significant increase in microhardness compared to the control group, indicating effective remineralization. SEM images revealed a new mineral layer formation on the enamel surface, and EDX analysis confirmed an increase in the calcium-to-phosphorus ratio, further validating remineralization. The study concluded that amine-terminated PAMAM dendrimers are slightly more effective in promoting the remineralization of artificial initial enamel caries. This research underscores the potential of PAMAM dendrimers as a promising agent for enamel caries treatment and prevention.

The study by Gao et al. [14] explored the enamel remineralization potential of PAMAM combined with adhesive resin containing calcium phosphate nanoparticles. The research focused on creating an effective treatment method for enamel caries by leveraging the synergistic effects of PAMAM dendrimers and calcium phosphate nanoparticles. Enamel specimens were artificially demineralized to simulate initial caries lesions and then treated with the PAMAM dendrimer and adhesive resin mixture. The remineralization effectiveness was assessed through microhardness testing and SEM-EDX. Microhardness tests indicated a substantial increase in enamel hardness, while SEM images showed the formation of a new mineral layer on the enamel surface. EDX analysis revealed an increased calcium-to-phosphorus ratio in the treated specimens, confirming effective remineralization. The study concluded that the combination of PAMAM dendrimers and adhesive resin containing calcium phosphate nanoparticles holds significant promise for enhancing enamel remineralization and presents a potential innovative approach for dental caries treatment.

The study by Khater et al. [15] investigated the effects of PAMAM, nanohydroxyapatite (nHA), and their combination on the microhardness of demineralized enamel. The research aimed to determine the most effective treatment for enhancing enamel hardness and promoting remineralization. Enamel specimens were artificially demineralized to simulate initial caries lesions and then divided into three treatment groups: PAMAM dendrimers, nHA, and a combination of both. The microhardness of the enamel specimens was measured before and after treatment using microhardness testing. Results indicated that all three treatments significantly improved enamel microhardness compared to the control group. The combination of PAMAM dendrimers and nHA showed the most pronounced increase in microhardness, suggesting a synergistic effect. The study concluded that both PAMAM dendrimers and nHA are effective in enhancing the microhardness of demineralized enamel, with their combination offering the greatest potential for enamel remineralization and caries treatment.

The study by Deokar et al. [16] conducted a comparative assessment of the remineralization potential of sodium fluoride and PAMAM on artificial caries-like lesions of enamel in vitro. The research aimed to evaluate and compare the effectiveness of these two agents in promoting enamel remineralization. Enamel specimens were artificially demineralized to create caries-like lesions and then divided into two treatment groups: one treated with NaF and the other with PAMAM dendrimers. The effectiveness of remineralization was evaluated using microhardness testing and SEM-EDX. Results showed that both NaF and PAMAM dendrimers significantly improved the microhardness of demineralized enamel compared to the control group. SEM images revealed the formation of a new mineral layer on the enamel surface in both treatment groups, while EDX analysis confirmed an increased calcium-to-phosphorus ratio, indicating effective remineralization. The study concluded that both NaF and PAMAM dendrimers are effective in promoting enamel remineralization, with PAMAM dendrimers showing a marginally superior performance. This research highlights the potential of PAMAM dendrimers as an alternative to traditional fluoride treatments for enamel caries prevention and repair.

The study by Meghana et al. [17] compared the enamel remineralization potential of PAMAM and CPP-ACPF in the primary teeth in vitro. The research aimed to evaluate the effectiveness of these two remineralizing agents on the demineralized enamel of the primary teeth. Human primary teeth specimens were artificially demineralized to create caries-like lesions and then divided into two treatment groups: one treated with PAMAM dendrimers and the other with CPP-ACPF. The remineralization effectiveness was assessed using microhardness testing and SEM-EDX. Results indicated that both PAMAM dendrimers and CPP-ACPF significantly increased the microhardness of demineralized enamel compared to the control group. However, PAMAM dendrimers showed a slightly higher increase in enamel hardness than CPP-ACPF. SEM images demonstrated the formation of a new mineral layer on the enamel surface for both treatment groups and EDX analysis revealed an improved calcium-to-phosphorus ratio, confirming effective remineralization. The study concluded that both PAMAM dendrimers and CPP-ACPF are effective in promoting enamel remineralization in the primary teeth, with PAMAM dendrimers exhibiting a marginally superior performance. This research underscores the potential of PAMAM dendrimers as an effective alternative to CPP-ACPF for the remineralization and treatment of enamel caries in the primary teeth.

Despite the promising results, several limitations should be considered. The heterogeneity among the included studies, in terms of study design, sample size, and assessment methods, poses a challenge in drawing definitive conclusions. Additionally, most studies have been conducted in vitro, and there is a need for well-designed clinical trials to validate these findings in vivo. Future research should also explore the long-term stability of the remineralized enamel and the safety profile of dendrimers.

## Conclusions

In conclusion, dendrimers exhibit significant potential as a novel remineralizing agent for enamel. Their unique structural properties and mechanism of action allow for effective and deep remineralization, positioning them as a promising alternative to conventional agents. However, further clinical research is warranted to fully elucidate their efficacy and safety in real-world settings. The integration of dendrimers into clinical practice could revolutionize the approach to enamel remineralization and caries prevention.
