# Correction: Examining the Effectiveness of Polyamidoamine (PAMAM) Dendrimers for Enamel Lesion Remineralization: A Systematic Review

**DOI:** 10.7759/cureus.c317

**Published:** 2025-09-24

**Authors:** Sri Meghana Sanka, Kavitha Ramar

**Affiliations:** 1 Pediatric and Preventive Dentistry, Sri Ramaswamy Memorial (SRM) Kattankulathur Dental College and Hospital, Chennai, IND; 2 Pedodontics and Preventive Dentistry, Sri Ramaswamy Memorial (SRM) Kattankulathur Dental College and Hospital, Chennai, IND

This article has been corrected to fix the following typo in the last sentence of the first paragraph in the Data Abstraction section: The number of articles excluded has been corrected from 3,695 to 3,675.

